# Longitudinal trajectories of spectral power during sleep in middle-aged and older adults

**DOI:** 10.1016/j.nbas.2022.100058

**Published:** 2022-12-16

**Authors:** Chenlu Gao, Michael K. Scullin

**Affiliations:** a Department of Psychology and Neuroscience, Baylor University, Waco, TX, USA; b Division of Sleep and Circadian Disorders, Departments of Medicine and Neurology, Brigham and Women’s Hospital, Boston, MA, USA; c Division of Sleep Medicine, Harvard Medical School, Boston, MA, USA; d Broad Institute of MIT and Harvard, Cambridge, MA, USA

**Keywords:** Sleep Heart Health Study, Sleep micro-architecture, Cognition, Dementia, Polysomnography

## Abstract

Age-related changes in sleep appear to contribute to cognitive aging and dementia. However, most of the current understanding of sleep across the lifespan is based on cross-sectional evidence. Using data from the Sleep Heart Health Study, we investigated longitudinal changes in sleep micro-architecture, focusing on whether such age-related changes are experienced uniformly across individuals. Participants were 2,202 adults (age_Baseline_ = 62.40 ± 10.38, 55.36 % female, 87.92 % White) who completed home polysomnography assessment at two study visits, which were 5.23 years apart (range: 4–7 years). We analyzed NREM and REM spectral power density for each 0.5 Hz frequency bin, including slow oscillation (0.5–1 Hz), delta (1–4 Hz), theta (4–8 Hz), alpha (8–12 Hz), sigma (12–15 Hz), and beta-1 (15–20 Hz) bands. Longitudinal comparisons showed a 5-year decline in NREM delta (p <.001) and NREM sigma power density (p <.001) as well as a 5-year increase in theta power density during NREM (p =.001) and power density for all frequency bands during REM sleep (ps < 0.05). In contrast to the notion that sleep declines linearly with advancing age, longitudinal trajectories varied considerably across individuals. Within individuals, the 5-year changes in NREM and REM power density were strongly correlated (slow oscillation: r = 0.46; delta: r = 0.67; theta r = 0.78; alpha r = 0.66; sigma: r = 0.71; beta-1: r = 0.73; ps < 0.001). The convergence in the longitudinal trajectories of NREM and REM activity may reflect age-related neural de-differentiation and/or compensation processes. Future research should investigate the neurocognitive implications of longitudinal changes in sleep micro-architecture and test whether interventions for improving key sleep micro-architecture features (such as NREM delta and sigma activity) also benefit cognition over time.

## Introduction

Since objective sleep assessment became available in the 20th century, researchers have investigated how, and why, sleep physiology changes during aging. [1], [2], [3] These basic science questions are important to translational, health issues. For example, because sleep is critical for cognition, age-related changes in sleep physiology may affect prefrontal cortex function, impede memory consolidation, or allow accumulation of metabolic products, each of which may contribute to cognitive decline and dementia. [4], [5], [6], [7], [8] Therefore, there is a need to understand how sleep typically changes with aging, including the rate of change at different ages, whether sleep changes consistently across individuals, and whether all aspects of sleep physiology change consistently within individuals (i.e., globally worsening versus some aspects of sleep declining and others showing preservation). [9].

The majority of the field’s knowledge on sleep and aging is built on cross-sectional studies. For example, Ohayon et al. (2004) meta-analyzed cross-sectional data from 65 studies with participants 5–102 years old and observed worsening in all sleep macro-architecture measures with aging, including linear decreases in total sleep time (TST), sleep efficiency (SE), slow-wave sleep (SWS), and rapid eye movement (REM) sleep; they also observed age-related increases in wake after sleep onset (WASO). [10] Similarly, a recent meta-analysis reported decreases in TST and SE, as well as increases in percentages of N1 sleep and WASO with aging. [11] Subsequent cross-sectional studies that compared younger and older adults have also shown these age-related macro-architectural changes in sleep. [12], [13], [14], [15], [16] Beyond macro-architecture, aging might compromise sleep micro-architecture and sleep-based electroencephalography (EEG) waveform characteristics. [17] Cross-sectionally, researchers have typically observed that aging compromises spindle density/duration, [18], [19] Non-REM (NREM) delta, [20], [21], [22], [23], [24], [25] NREM theta, [21], [24], [25], [26], [27] and NREM sigma spectral power. [23], [26] Furthermore, though age-related changes in REM spectral power are less studied, cross-sectional evidence suggests decreases in lower frequency activities and increases in higher frequency activities with aging. [27], [28], [29].

Longitudinal studies have indicated a more complex picture of sleep changes with aging. For example, macro-architecture analyses on participants in the longitudinal Sleep Heart Health Study (SHHS) revealed great inter-individual variability in how sleep changed over time; some participants’ macro-architecture worsened, some stayed the same, and some even improved. [9] Another noteworthy finding was that though cross-sectional analyses suggested a reduction in REM sleep with aging (consistent with prior cross-sectional research), the longitudinal data showed a 3-minute increase in REM sleep over 5 years. [9] This discrepancy raised the possibility that sleep and aging cross-sectional studies could be influenced by cohort effects or related biases; or, that NREM and REM sleep physiology change dynamically within individuals across time. In support of the latter view, longitudinal changes in SWS and REM sleep minutes were weakly associated, with patterns suggesting that an increase in REM sleep over time might compensate for a reduction in SWS in some individuals.

Dynamic changes in NREM and REM sleep with aging have received minimal attention. More often, researchers have investigated age effects on NREM and REM sleep independently, perhaps because the two sleep stages rely on different neural mechanisms. [30], [31], [32] As Carskadon and Dement put it, NREM and REM sleep have been considered “as distinct from one another as each is from wakefulness.” [33] Such conclusions have largely been based on studies with young animal models or healthy young adults. Recent theories of neurocognitive aging indicate that aging is accompanied by neural de-differentiation, during which neuronal mechanisms lose their specialization. [34] For example, the visual cues that are associated with activation in specific brain regions in young adults are associated with much wider-spread activation in older adults. [35] Furthermore, as aging brains lose their specialization, there is a corresponding decline in memory functioning. [36] These general neurobiological outcomes may have relevance to understanding how NREM and REM sleep interact during the aging process to influence cognitive outcomes (e.g., the sequential hypothesis of memory processing during sleep). [37], [38], [39], [40].

In summary, current understanding of age-related changes in sleep physiology is mainly based on cross-sectional evidence of relatively small convenience samples. Data from large, longitudinal studies will better inform how sleep physiology changes with aging. Moreover, it remains unclear whether the neural mechanisms that generate NREM and REM sleep also undergo age-related de-differentiation. Therefore, the current study aims to inform these gaps in the literature by investigating longitudinal changes in NREM and REM sleep micro-architecture in a large sample of middle-to-older aged adults across 5 years.

## Methods

Spectral power data were available for 2,202 participants who completed two study visits in SHHS 4–7 years apart (55.36 % female; 87.92 % White). [41] SHHS is a multi-center longitudinal study that recruited individuals ≥ 40 years old without prior treatment for sleep-disordered breathing (Age_Visit1_: Range = 39–86; M ± SD = 62.40 ± 10.38; Age_Visit2_: Range = 44–90; M ± SD = 67.63 ± 10.30). This sample was originally recruited to investigate cardiovascular consequences of sleep-disordered breathing, with approximately 6 % of them reporting consuming ≥ 2 alcoholic drinks/day, 11 % being current smokers, and 17 % diagnosed with cardiovascular disease at baseline. [42] Visit 1 data collection occurred between 1995 and 1998 and Visit 2 occurred between 2001 and 2003.

Detailed methods for the SHHS were previously published. [41] In summary, one-night home polysomnography was collected at each visit via EEG (C3-A2 and C4-A1 channels), electrooculography, chin electromyography, thoracic and abdominal bands, nasal-oral thermocouples, finger pulse oximetry, and bipolar electrocardiography. Spectral power density was calculated for each 0.5 Hz bin for 0.5 Hz-25 Hz and for slow oscillation (0.5–1 Hz), delta (1–4 Hz), theta (4–8 Hz), alpha (8–12 Hz), sigma (12–15 Hz), and beta-1 (15–20 Hz), separated by REM and NREM sleep stages. Because only existing de-identified data were used in this study, approval from the university Institutional Review Board was waived.

Prior to statistical analyses, power density values were normalized via log-transformation. [43] We additionally excluded outliers that were > 1.5 interquartile ranges from the 1st/3rd quartile. [44] Cross-sectionally, we conducted Pearson’s correlations to test the association between age, macro-architecture, and micro-architecture of sleep at Visit 1. Longitudinally, we conducted paired-sample t-tests to assess intra-individual changes in sleep and Pearson’s correlations to determine the relationships between NREM and REM power density. We repeated the analyses in males (n = 983) and females (n = 1,219) separately and in middle-aged (age_visit1_ ≤ 60; n = 984) and older participants separately (age_visit1_ > 60; n = 1218). Statistical analyses (two-tailed, alpha = 0.05) were performed using SPSS 28 and figures were generated using SAS 9.4 and GraphPad Prism 9.

## Results

### Associations between macro- and micro-architecture

Participants’ macro- and micro-architecture sleep characteristics at both visits are summarized in Table 1. Most correlations between sleep micro-architecture and macro-architecture were small-sized (Table 2). For example, greater NREM power density in the 0.5–15 Hz bands correlated with better sleep macro-architecture, including longer TST, shorter WASO, and higher SE (largest *r* = 0.15, *p* <.001). By contrast, greater NREM power density in beta-1 band correlated with shorter TST (*r* = -0.08, *p* <.001) and longer WASO (*r* = 0.04, *p* =.049). Moreover, greater REM delta and REM theta power density correlated with better sleep macro-architecture (e.g., longer TST and lower WASO) whereas greater REM power density in slow oscillation, sigma, and beta-1 bands correlated with poorer sleep (e.g., shorter TST, longer WASO, and lower SE; Table 2).

**Table 1:** Participants’ age and sleep characteristics.

	Visit 1	Visit 2	Longitudinal change (p-value, Cohen’s d)
Age	62.40 (10.38)	67.63 (10.30)	N/A
Stage 1 sleep (min)	18.02 (12.71)	20.32 (15.54)	p <.001, d = 0.14***
Stage 2 sleep (min)	210.32 (55.07)	215.37 (56.68)	p <.001, d = 0.08***
Slow wave sleep (min)	67.05 (43.28)	61.51 (42.06)	p <.001, d = 0.16***
REM sleep (min)	75.62 (28.03)	78.51 (30.22)	p <.001, d = 0.08***
Total sleep time (min)	371.02 (58.68)	375.67 (68.89)	p =.003, d = 0.06**
Sleep onset latency (min)^a^	21.00 (20.16)	26.19 (29.55)	p <.001, d = 0.16***
Wake after sleep onset (min)	57.52 (39.67)	79.96 (54.52)	p <.001, d = 0.40***
Sleep efficiency (%)^a^	83.58 (8.92)	79.61 (11.50)	p <.001, d = 0.33***
**NREM spectral power density (log-transformed)**^b^			
Slow oscillation (0.5–1 Hz)	2.01 (0.22)	2.00 (0.22)	p =.145, d = 0.03
Delta (1–4 Hz)	1.51 (0.18)	1.49 (0.19)	p <.001, d = 0.14***
Theta (4–8 Hz)	0.87 (0.19)	0.88 (0.20)	p =.001, d = 0.07***
Alpha (8–12 Hz)	0.61 (0.22)	0.60 (0.22)	p =.358, d = 0.02
Sigma (12–15 Hz)	0.31 (0.21)	0.29 (0.21)	p <.001, d = 0.24***
Beta-1 (15–20 Hz)	−0.15 (0.18)	−0.16 (0.18)	p =.201, d = 0.03
**REM spectral power density (log-transformed)**^b^			
Slow oscillation (0.5–1 Hz)	1.46 (0.18)	1.49 (0.19)	p <.001, d = 0.16***
Delta (1–4 Hz)	1.02 (0.15)	1.03 (0.16)	p <.001, d = 0.09***
Theta (4–8 Hz)	0.60 (0.19)	0.63 (0.20)	p <.001, d = 0.19***
Alpha (8–12 Hz)	0.34(0.22)	0.37 (0.23)	p <.001, d = 0.21***
Sigma (12–15 Hz)	0.06 (0.21)	0.08 (0.21)	p <.001, d = 0.17***
Beta-1 (15–20 Hz)	−0.163 (0.21)	−0.156 (0.21)	p =.024, d = 0.05*

Note. Values are presented as mean (SD).

*p <.05, **p <.01, ***p <.001.

a Sleep onset latency and sleep efficiency were only available and deemed reliable in 1,022 participants.

b Spectral power density values were log-transformed to improve normality.

**Table 2:** Correlations between sleep macro- and micro-architecture at Visit 1.

	Total sleep time	Wake after sleep onset	Sleep onset latency	Sleep efficiency
NREM spectral power density				
Slow oscillation (0.5–1 Hz)	r = 0.05, p =.023*	r = -0.15, p <.001***	r = 0.01, p =.790	r = 0.09, p =.003**
Delta (1–4 Hz)	r = 0.05, p =.019*	r = -0.14, p <.001***	r = -0.02, p =.637	r = 0.09, p =.006**
Theta (4–8 Hz)	r = 0.02, p =.260	r = -0.08, p <.001***	r = -0.01, p =.656	r = 0.08, p =.018*
Alpha (8–12 Hz)	r = -0.02, p =.401	r = -0.05, p =.011*	r = -0.02, p =.476	r = 0.06, p =.053
Sigma (12–15 Hz)	r = 0.01, p =.791	r = -0.07, p =.001**	r = -0.04, p =.171	r = 0.07, p =.023*
Beta-1 (15–20 Hz)	r = -0.08, p <.001***	r = 0.04, p =.049*	r = -0.02, p =.622	r = -0.03, p =.300
REM spectral power density				
Slow oscillation (0.5–1 Hz)	r = -0.02, p =.506	r = 0.03, p =.119	r = 0.03, p =.349	r = -0.10, p =.001**
Delta (1–4 Hz)	r = 0.04, p =.049*	r = -0.08, p <.001***	r = 0.02, p =.619	r = 0.02, p =.531
Theta (4–8 Hz)	r = 0.01, p =.652	r = -0.07, p =.001**	r = 0.01, p =.848	r = 0.04, p =.208
Alpha (8–12 Hz)	r = -0.07, p <.001***	r = 0.02, p =.349	r = 0.03, p =.420	r = -0.03, p =.278
Sigma (12–15 Hz)	r = -0.11, p <.001***	r = 0.05, p =.016*	r = -0.02, p =.524	r = -0.04, p =.210
Beta-1 (15–20 Hz)	r = -0.10, p <.001***	r = 0.06, p =.006**	r = -0.02, p =.571	r = -0.05, p =.153

*p <.05, **p <.01, ***p <.001.

## Cross-sectional analyses of the sleep power spectrum and aging

Cross-sectionally, at Visit 1, older age correlated with lower NREM delta (*r* = -0.12, *p* <.001) and sigma activity (*r* = -0.22, *p* <.001; all other bands, *p*s > 0.05; Table 3). In males, older age correlated with lower NREM power in all frequency bands (*r*s between −0.31 and −0.12, *p*s < 0.001). By contrast, in females, older age only correlated with lower sigma power (*r* = -0.15, *p* <.001); interestingly, older females showed slightly *increased* NREM theta and beta-1 power (*r*s = 0.07, *p*s < 0.05). Table 3 further indicates that some changes with aging were non-linear: during middle-age, NREM alpha and beta-1 power increased with age, but during older age, NREM alpha and beta-1 power decreased with aging.

**Table 3:** Correlations between age and NREM and REM spectral power density.

	All participants	Males	Females	Middle-aged (≤60 years old)	Older (>60 years old)
**Correlations between age and NREM power density at Visit 1**					
Slow oscillation (0.5–1 Hz)	r = -0.04, p =.094	r = -0.16, p <.001***	r = 0.05, p =.080	r = 0.02, p =.467	r = 0.02, p =.570
Delta (1–4 Hz)	r = -0.12, p <.001***	r = -0.26, p <.001***	r = -0.04, p =.205	r = 0.003, p =.932	r = -0.05, p =.082
Theta (4–8 Hz)	r = -0.01, p =.671	r = -0.12, p <.001***	r = 0.07, p =.019*	r = 0.04, p =.182	r = 0.03, p =.265
Alpha (8–12 Hz)	r = -0.04, p =.085	r = -0.13, p <.001***	r = 0.03, p =.336	r = 0.08, p =.009**	r = -0.07, p =.023*
Sigma (12–15 Hz)	r = -0.22, p <.001***	r = -0.31, p <.001***	r = -0.15, p <.001***	r = 0.04, p =.186	r = -0.19, p <.001***
Beta-1 (15–20 Hz)	r = -0.02, p =.272	r = -0.14, p <.001***	r = 0.07, p =.015*	r = 0.08, p =.009**	r = -0.07, p =.015*
**Correlations between age and REM power density at Visit 1**					
Slow oscillation (0.5–1 Hz)	r = 0.07, p =.001**	r = 0.05, p =.142	r = 0.09, p =.002**	r = 0.08, p =.010*	r = -0.01, p =.696
Delta (1–4 Hz)	r = -0.01, p =.647	r = -0.12, p <.001***	r = 0.07, p =.011*	r = 0.06, p =.080	r = -0.03, p =.270
Theta (4–8 Hz)	r = 0.04, p =.056	r = -0.06, p =.057	r = 0.13, p <.001***	r = 0.06, p =.067	r = 0.04, p =.215
Alpha (8–12 Hz)	r = 0.15, p <.001***	r = 0.05, p =.127	r = 0.23, p <.001***	r = 0.09, p =.007**	r = 0.06, p =.038*
Sigma (12–15 Hz)	r = 0.16, p <.001***	r = 0.02, p =.500	r = 0.28, p <.001***	r = 0.18, p <.001***	r = 0.003, p =.919
Beta-1 (15–20 Hz)	r = 0.10, p <.001***	r = -0.04, p =.178	r = 0.22, p <.001***	r = 0.17, p <.001***	r = -0.04, p =.154
**Correlations between age and change in NREM power density**					
Slow oscillation (0.5–1 Hz)	r = -0.03, p =.252	r = 0.03, p =.333	r = -0.07, p =.012*	r = -0.12, p <.001***	r = 0.07, p =.014*
Delta (1–4 Hz)	r = -0.10, p <.001***	r = -0.01, p =.837	r = -0.16, p <.001***	r = -0.13, p <.001***	r = 0.01, p =.753
Theta (4–8 Hz)	r = -0.04, p =.086	r = 0.03, p =.351	r = -0.09, p =.002**	r = -0.12, p <.001***	r = 0.06, p =.026*
Alpha (8–12 Hz)	r = -0.16, p <.001***	r = -0.10, p =.002**	r = -0.21, p <.001***	r = -0.19, p <.001***	r = 0.03, p =.286
Sigma (12–15 Hz)	r = -0.10, p <.001***	r = -0.08, p =.009**	r = -0.11, p <.001***	r = -0.18, p <.001***	r = 0.07, p =.013*
Beta-1 (15–20 Hz)	r = -0.07, p =.002**	r = -0.07, p =.040*	r = -0.07, p =.022*	r = -0.05, p =.130	r = 0.04, p =.218
**Correlations between age and change in REM power density**					
Slow oscillation (0.5–1 Hz)	r = -0.004, p. = 849	r = 0.02, p =.487	r = -0.03, p =.381	r = -0.07, p =.040*	r = 0.05, p =.103
Delta (1–4 Hz)	r = 0.003, p =.907	r = 0.07, p =.045*	r = -0.05, p =.103	r = -0.07, p =.038	r = 0.10, p =.001**
Theta (4–8 Hz)	r = 0.03, p =.241	r = 0.08, p =.016*	r = -0.02, p =.619	r = -0.11, p =.001**	r = 0.11, p <.001***
Alpha (8–12 Hz)	r = 0.04, p =.057	r = 0.05, p =.136	r = 0.04, p =.214	r = -0.07, p =.023*	r = 0.14, p <.001***
Sigma (12–15 Hz)	r = -0.08, p <.001***	r = -0.08, p =.017*	r = -0.08, p =.008**	r = -0.12, p <.001***	r = 0.05, p =.091
Beta-1 (15–20 Hz)	r = -0.13, p <.001***	r = -0.11, p =.001**	r = -0.15, p <.001***	r = -0.12, p <.001***	r = 0.003, p =.925

Note. Spectral power density values were log-transformed to improve normality. For changes in NREM/REM power density, positive values represent increases from Visit 1 to Visit 2; negative values represent decreases from Visit 1 to Visit 2.

*p <.05, **p <.01, ***p <.001.

REM sleep spectral power showed different outcomes. Cross-sectionally, older age correlated with greater REM power density in slow oscillations (*r* = 0.07, *p* =.001), alpha (*r* = 0.15, *p* <.001), sigma (*r* = 0.16, *p* <.001), and beta-1 bands (*r* = 0.10, *p* <.001; but not theta or delta, *p*s > 0.05). Such increases in power density were driven by females (*r*s ≥ 0.07, *p*s < 0.05 for all frequency bands), whereas males showed no increases in REM power in older age (*p*s > 0.05, or even a decrease in delta power, *r* = -0.12, *p* <.001). Furthermore, analyses separated by age groups suggested that the increases in REM power with aging were more prominent amongst middle-aged adults (slow oscillation: *r* = 0.08, alpha: *r* = 0.09, sigma: *r* = 0.18; beta-1: *r* = 0.17, *p*s < 0.05), with attenuation amongst older adults (all bands: *r*s < 0.05, *p*s > 0.05, except for alpha: *r* = 0.06, *p* =.038).

## Longitudinal changes in sleep micro-architecture

**Fig. 1:**
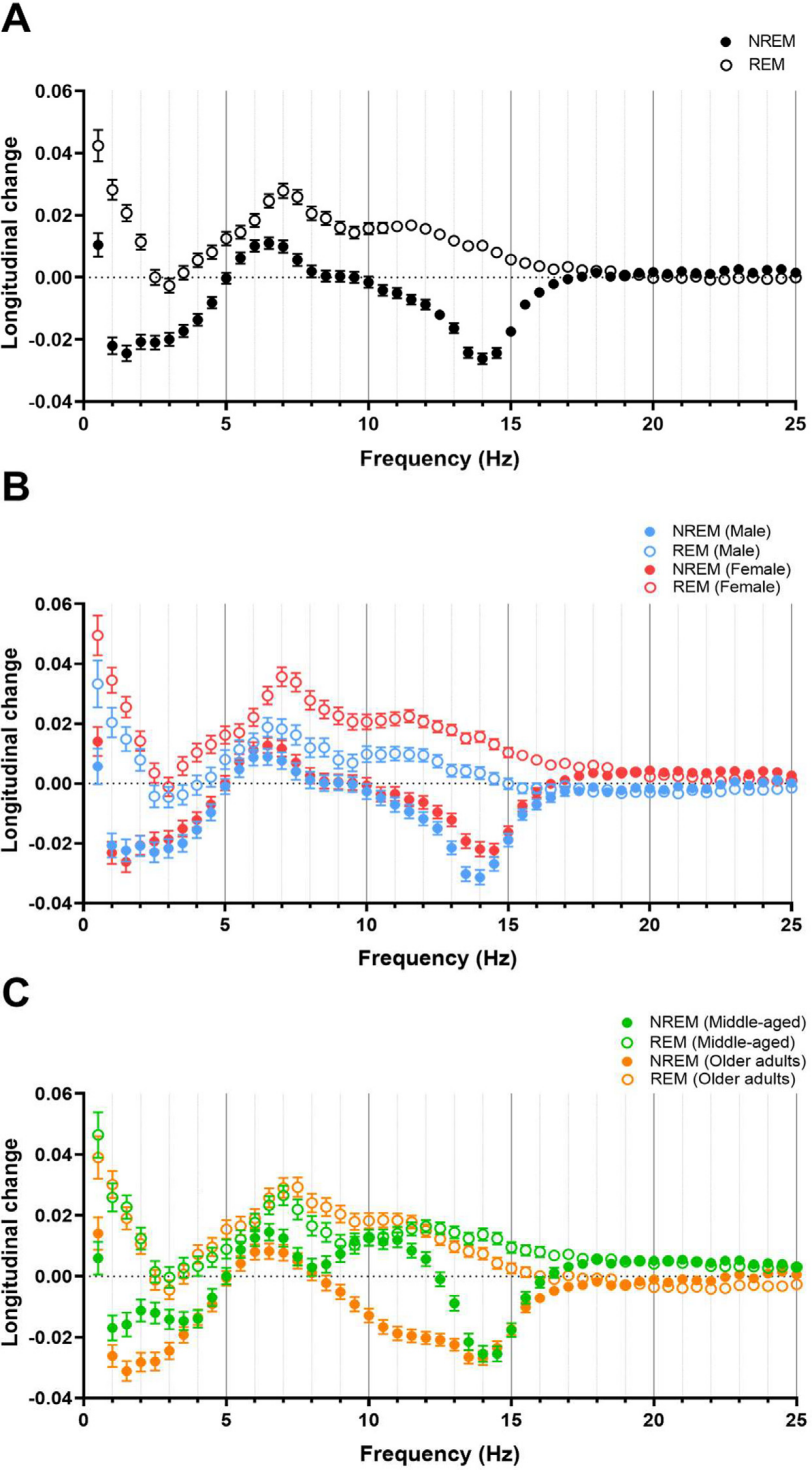
Changes in log-transformed NREM and REM spectral power density from Visit 1 to Visit 2 in all participants (A), in males and females separately (B), and in middled-aged and older adults separately (C). Positive values indicate increases in spectral power density and negative values indicate decreases in spectral power density from Visit 1 to Visit 2. Error bars represent standard error.

**Fig. 2:**
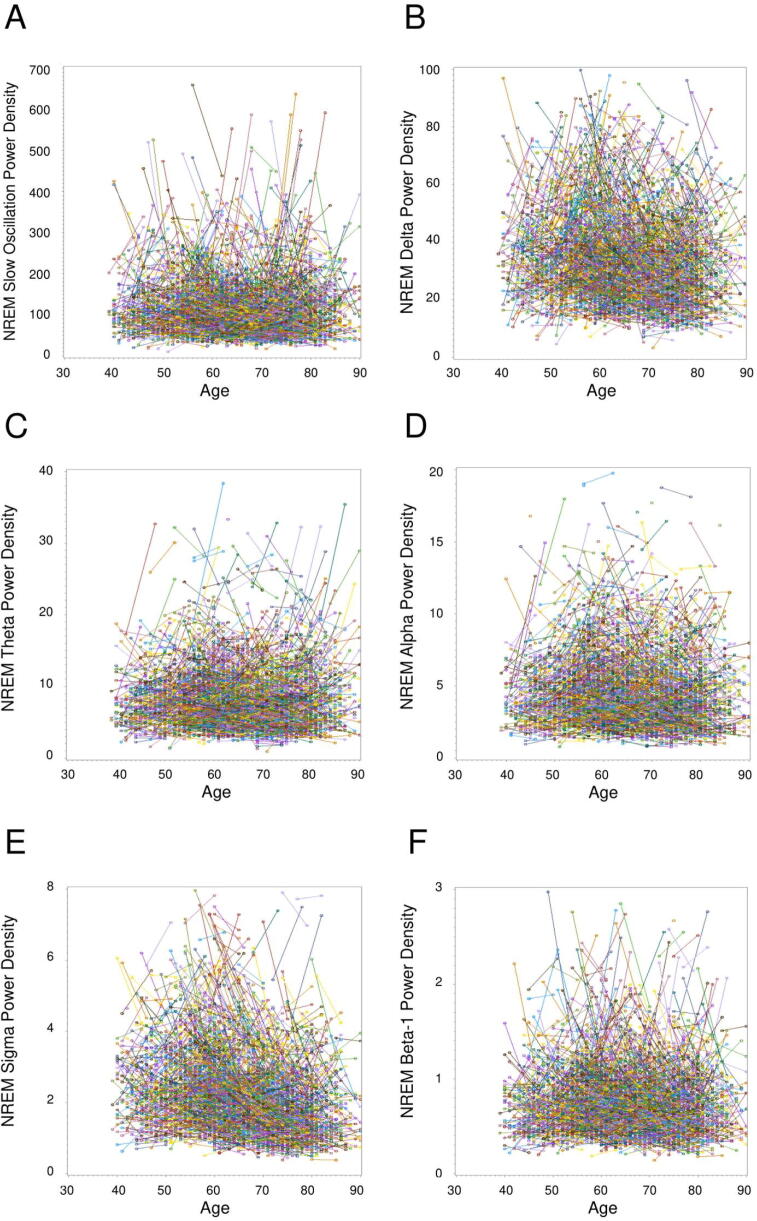
NREM sleep individual longitudinal trajectories in slow oscillation (A), delta (B), theta (C), alpha (D), sigma (E), and beta-1 (F) spectral power density (μV^2^/Hz). Each line represents the changes in spectral power density in one participant from Visit 1 to Visit 2. Visually identified outliers (n < 10 per figure) were not included in the figures.

Fig. 1, Fig. 2 illustrate the longitudinal changes in NREM power density. On average, NREM power density significantly decreased from Visit 1 to Visit 2 in delta and sigma bands (*p*s < 0.001; Table 1), converging with cross-sectional reports in the literature of age-related declines in slow-wave activity and spindles. [18], [23] By contrast, NREM theta power density significantly increased (*p* =.001; no changes in slow oscillations, alpha, or beta-1, *p*s > 0.05). Consistent with cross-sectional observations and implicating a nonlinear change in sleep, NREM alpha power density increased longitudinally amongst middle-aged adults (*p* <.001) but decreased longitudinally amongst older adults (*p* <.001).

**Fig. 3:**
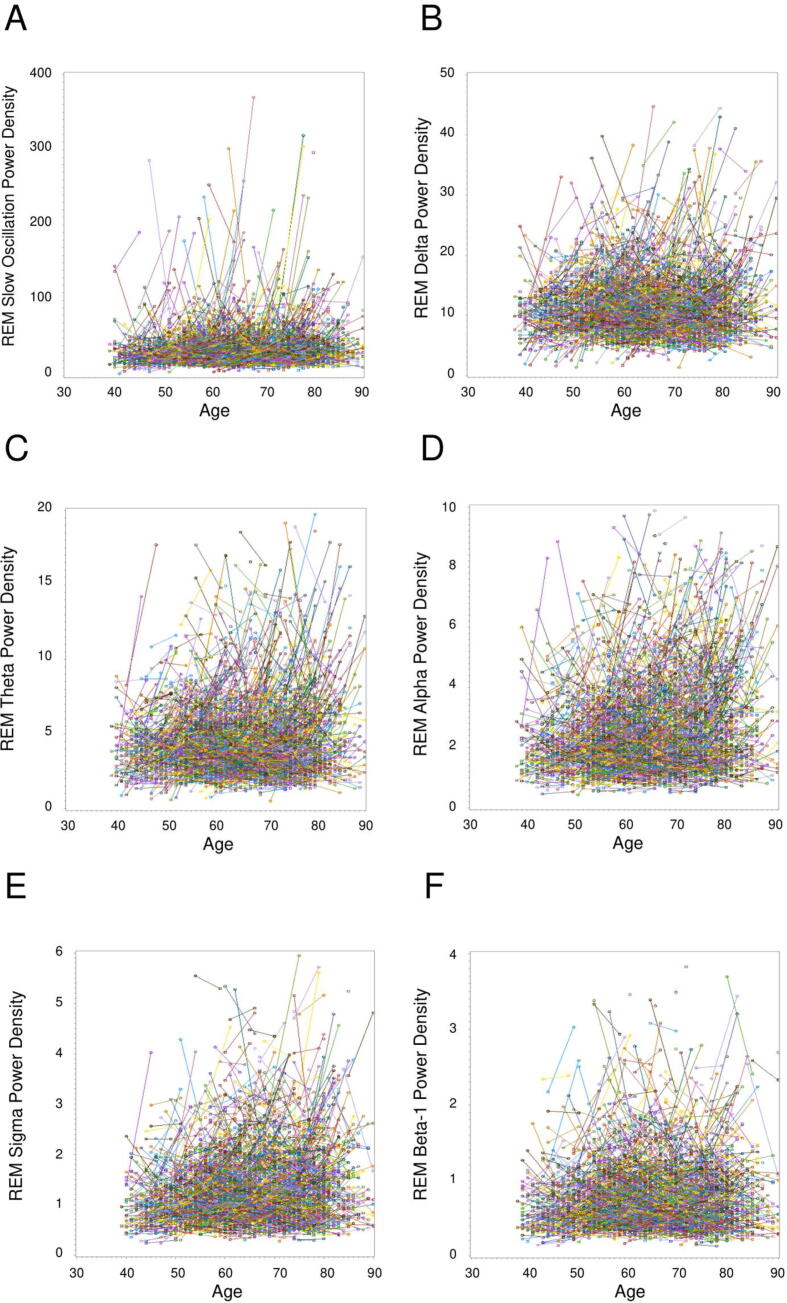
REM sleep individual longitudinal trajectories in slow oscillation (A), delta (B), theta (C), alpha (D), sigma (E), and beta-1 (F) spectral power density (μV^2^/Hz). Each line represents the changes in spectral power density in one participant from Visit 1 to Visit 2. Visually identified outliers (n < 10 per figure) were not included in the figures.

A different longitudinal pattern emerged for REM sleep (Fig. 1, Fig. 3). REM power density increased for all bands (*p*s < 0.05; Table 1). Inspection of inter-individual longitudinal patterns in Fig. 2, Fig. 3 illustrate considerable individual differences in how NREM and REM power density changed over time. For example, females exhibited longitudinal increases in REM power density across all frequency bands (*p*s < 0.001); in males, such increases were limited to the slow oscillation (*p* =.002), theta (*p* <.001), and alpha bands (*p* <.001).

## Longitudinal correspondence between NREM and REM sleep features

**Fig. 4:**
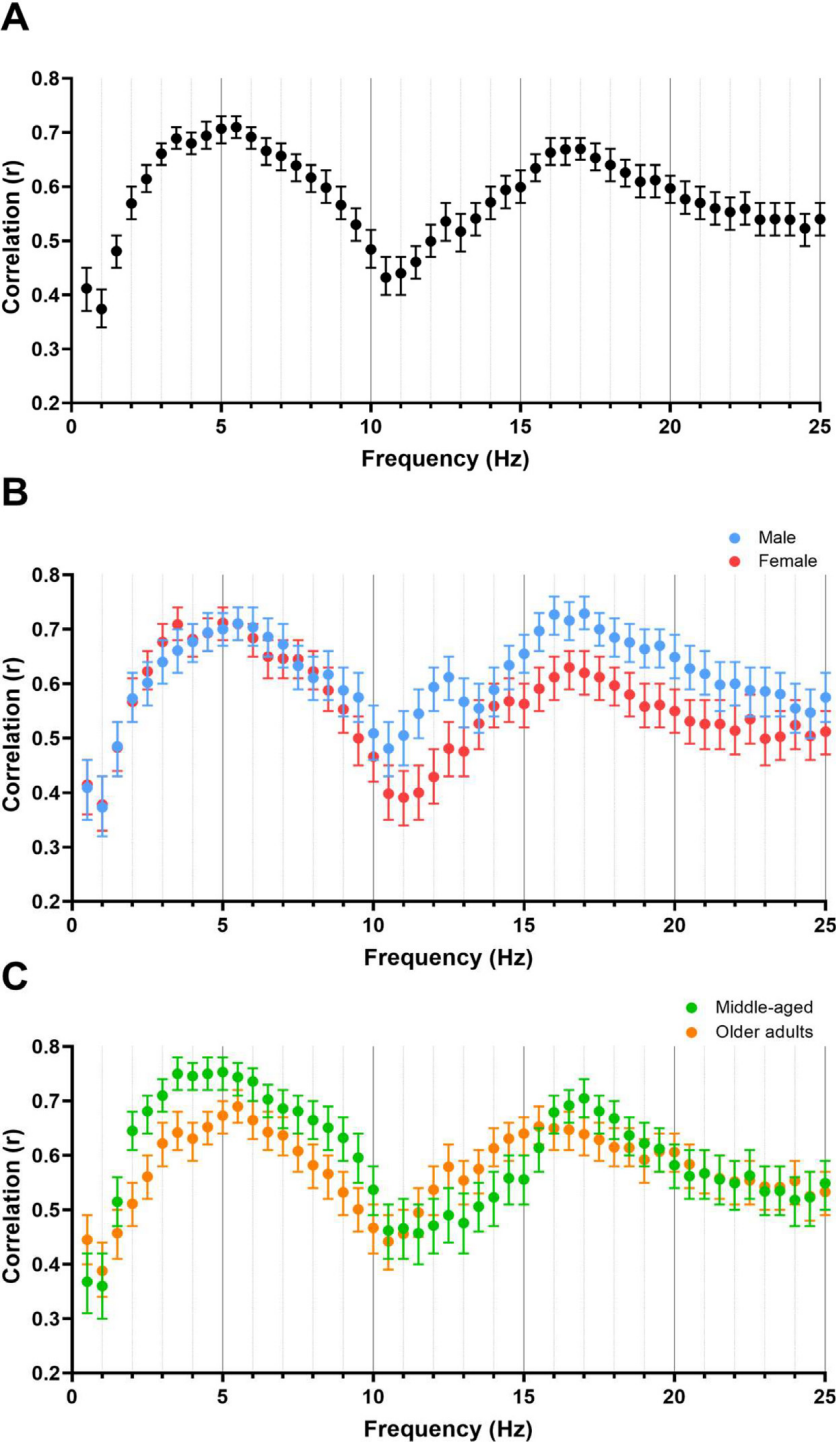
Correlation between changes in log-transformed NREM spectral power density and changes in log-transformed REM spectral power density from Visit 1 to Visit 2 by frequency in all participants (A), in males and females separately (B), and in middle-aged and older adults separately (C). Error bars represent 95% confidence intervals, which were based on Fisher's transformation.

We next examined the degree to which longitudinal changes in spectral power bands corresponded across NREM and REM stages. These stages have classically been conceptualized as qualitatively different brain states, with unique neural underpinnings. Interestingly though, Fig. 4 illustrates that longitudinal changes in NREM power density correlated strongly with changes in REM power density. While the NREM-REM longitudinal association was modest for slow oscillations (*r* = 0.46), the other power bands displayed very large correlations (delta: *r* = 0.67; theta *r* = 0.78; alpha *r* = 0.66; sigma: *r* = 0.71; beta-1 bands: *r* = 0.73; all *p*s < 0.001). Fig. 4b shows that the NREM-REM correlations were sometimes stronger in males than females, particularly for the alpha (males: *r*[95 %CI] = 0.71[0.67–0.74], *p* <.001; females: *r*[95 %CI] = 0.62[0.58–0.65], *p* <.001) and beta-1 bands (males: *r*[95 %CI] = 0.77[0.75–0.80], *p* <.001; females: *r*[95 %CI] = 0.68[0.65–0.71], *p* <.001). Moreover, Fig. 4c shows that the correlations were stronger in middle-aged than older participants in the delta (middle-aged: *r*[95 %CI] = 0.73[0.70–0.76], *p* <.001; older adults: *r*[95 %CI] = 0.62[0.58–0.65], *p* <.001) and theta bands (middle-aged: *r*[95 %CI] = 0.82[0.80–0.84], *p* <.001; older adults: *r*[95 %CI] = 0.76[0.73–0.78], *p* <.001).

## Discussion

Longitudinal investigations of sleep changes with aging can produce different outcomes than cross-sectional approaches. While both cross-sectional and longitudinal analyses showed that NREM delta and sigma power decreased with aging, longitudinal analyses showed increases in NREM theta power and REM delta power. Moreover, whereas cross-sectional approaches can sometimes lead to simplified interpretations that sleep changes uniformly with advancing age, we observed evidence for non-linear changes in aging and substantial inter-individual variability in the longitudinal trajectories of sleep micro-architecture. Some of this variability may be explained by gender differences in sleep micro-architecture, and some may be explained by neural de-differentiation and compensatory mechanisms.

Our observations of a decline in NREM delta [20], [21], [22], [23], [24], [25] and sigma power density [23], [26] with aging are consistent with past studies. The decline in delta activity has previously been reported to be asymmetrical with the left centro-parietal region showing attenuated age-related decline, potentially explained by less gray matter atrophy in the dominant hemisphere (in right-handed participants). [24] Moreover, Carrier et al. (2011) found that aging is not only associated with lower slow-wave density, but also lower slow-wave amplitude and longer slow-wave positive and negative phase durations, suggesting lengthening of depolarization and hyperpolarization phases at the cellular level. [45] A decline in sigma power could be an indicator of a decrease in sleep spindles (i.e., reductions in spindle number, density, and duration). [18] These changes could be due to changes in the GABAergic mechanisms in the thalamocortical and intracortical circuitry, as well as reduced cortical volume. [18], [46].

Our observation of non-linear changes for some measures (e.g., NREM alpha power) is a distinctive finding. Most cross-sectional studies of sleep and aging have implicated linear changes, [10] but a greater precision of trajectories with aging is afforded by longitudinal studies than in extreme-group (younger versus older) cross-sectional studies. One potential explanation is a non-linear degeneration of the thalamocortical circuitry that is responsible for generating alpha rhythms in the older adult group. [47] For example, attenuated alpha activity was observed during both wakefulness and NREM sleep in Alzheimer’s disease patients, [48], [49] and with advancing age there is increased incidence of undiagnosed cognitive impairment.

Another divergent finding was the observation of a longitudinal increase in NREM theta power, which was contrary to past cross-sectional findings. [21], [24], [25], [26], [27] The divergent results could be explained by differences in study designs (cross-sectional [21], [24], [25], [26], [27] vs longitudinal in SHHS), sleep environment (sleep laboratory [21], [24], [25], [26], [27] vs at-home in SHHS), age range of the sample (up to 65 years old [21], [24], [25], [26], [27] vs up to 90 years old in SHHS), and inclusion criteria (excluded [24], [25], [26], [27] vs included individuals with sleep apnea in SHHS). Additionally, patterns of age-related change in NREM theta power may depend on topography. [24], [27] For example, Sprecher et al. (2016) observed that the age-related decline in NREM theta power was limited to frontal regions, and in the current study, only central regions were measured. [24] Beyond these methodological considerations, we observed lower NREM theta power to be associated with greater WASO and lower SE, though insomnia has been previously observed to be associated with increased NREM relative theta power. [50] Future research should clarify the associations between sleep macro- and micro-architecture to further our understanding of longitudinal changes in NREM theta activity.

The outcomes for REM sleep are also worth careful consideration. Cross-sectional studies suggested an age-related decrease in lower frequency activity during REM, [27], [28], [29] whereas we observed longitudinal increases in REM power density in all frequency bands. The development of mild cognitive impairment and/or Alzheimer’s disease in some individuals, which has been associated with slowing of REM spectral power, [49], [51], [52] may drive the increases in lower frequency REM activity. Furthermore, the changes in REM sleep activity were primarily driven by females. While gender differences have been found cross-sectionally in sleep micro-architecture, [25], [53], [54], [55] the observation of gender differences in longitudinal trajectories of sleep micro-architecture is less documented. One potential explanation is that middle-aged females show thicker cortices in occipital, posterior cingulate, precentral, and postcentral regions than middle-aged males [56] and cortical thinning leads to a decline in NREM and REM delta activity. [57] It is also possible that the gender differences were not due to sleep physiology, but instead influenced by anatomical differences (e.g., skull thickness), [53] physiological differences (e.g., menopausal status), [58] or mental health differences (e.g., depression and anxiety). [59], [60].

In addition to the influence of gender, we observed great inter-individual variability in how sleep micro-architecture changed over time. Such variability across people in longitudinal trajectories is contrary to the longstanding thinking of uniform and unidirectional changes in sleep, and may indicate age-related neural de-differentiation [34] or compensatory mechanisms. [61] By the neural de-differentiation view, the high concordance in how NREM and REM EEG activity changes longitudinally is due to a de-segregation of neural mechanisms that were separately activated during NREM and REM sleep. By the compensation view, the strong correlations may indicate that additional neural systems are recruited in middle or older age to support hypothalamic or thalamocortical circuitry (and other typical generators of NREM and REM sleep) in compensation for the age-related cellular loss and altered synaptic connectivity in these key regions. [61], [62], [63] In our study, compared to middle-aged adults, older adults showed an attenuation in the longitudinal concordance between NREM and REM activity in delta and theta bands, which may reflect compromised ability to compensate in the oldest age groups. Future studies could test the role of compensatory mechanisms by linking neurocognitive outcomes with the degree of NREM and REM concordance.

Another potential explanation for the differences between age groups in NREM-REM correspondence may be the neural mechanisms related to insomnia. For example, Wu et al. studied the cross-sectional correlations between EEG spectral power during eye-closed wakefulness and NREM sleep among good sleeper controls and primary insomnia patients. [64] Compared to the controls, the insomnia group showed weaker correlations in the delta, theta, and alpha bands, but a stronger correlation in the sigma band. Together with Wu et al.’s findings, our findings suggest that the mechanisms underlying delta and theta activity (e.g., regulation of homeostatic sleep drive [65] and sensory stimuli gating [66]) degenerate as a consequence of aging or primary insomnia.

The aging effect on sleep physiology has implications for age-related cognitive decline and dementia. [67] For example, EEG features during NREM and REM sleep are believed to reflect cortical integrity [57] and one’s brain age. [68] Such structural changes in the brain may exert broad influences, including moderating the memory consolidation effects of sleep, [69] thereby causing the memory outcomes to be correlated differently with sleep across younger and older adults. [70], [71] In some cases, cognitive processes may correlate with sleep physiology even in an opposite manner across younger and older brains. [72] According to the sequential hypothesis, NREM and REM collaboratively contribute to memory consolidation. [37], [38] Therefore, studying neural de-differentiation and compensation across NREM and REM sleep physiology will inform age-related cognitive decline and dementia.

Limitations of the current study include a lack of cognitive outcome data in the SHHS. The small proportion (12 %) of non-White participants also limits the investigation of racial/ethnic differences, or how sleep-memory trajectories may differ with aging in disadvantaged/marginalized groups. [73], [74] In the SHHS, EEG data were only collected from the central channels and first-night effects could influence some outcomes (though such effects are often absent in home polysomnography studies). [76] Future studies with adaptation nights and more EEG channels will inform the EEG topography of the aging effects [24], [27] and applying multitaper spectral analysis may illuminate age-related changes in a manner that Fourier analyses do not. [17], [75].

In conclusion, in this large, longitudinal sample, NREM power decreased in delta and sigma bands and that REM power increased in nearly all frequency bands with aging. There were sizeable individual differences in how EEG micro-architecture changed over 5 years, which may be influenced by gender differences, neural de-differentiation, and/or compensatory mechanisms. Future studies should investigate whether the changes in spectral power features precipitate cognitive decline and predict neurodegenerative diseases (or vice versa). There is also a need for longitudinal randomized controlled trials to determine whether interventions can durably change sleep physiology with advancing age; and if so, whether such changes in sleep can durably benefit cognitive functioning.

## CRediT authorship contribution statement

**Chenlu Gao:** Formal analysis, Methodology, Visualization, Writing – original draft. **Michael K. Scullin:** Conceptualization, Methodology, Resources, Writing – review & editing.

## Declaration of Competing Interest

The authors declare that they have no known competing financial interests or personal relationships that could have appeared to influence the work reported in this paper.
